# Botulinum toxin in the treatment of trigeminal neuralgia: 6-Month follow-up

**DOI:** 10.1097/MD.0000000000008133

**Published:** 2017-09-29

**Authors:** Ülkü Türk Börü, Arda Duman, Cem Bölük, Sanem Coşkun Duman, Mustafa Taşdemir

**Affiliations:** a Department of Neurology, Dr. Lütfi Kırdar Kartal Training and Research Hospital; b Department of Neurology, Maltepe State Hospital; c Department of Public Health, Bezmialem University, Istanbul, Turkey.

**Keywords:** botulinum toxin, treatment, trigeminal neuralgia

## Abstract

**Background:**

Botulinum toxin type-A (BTX-A) has been successfully utilized to treat trigeminal neuralgia. In this study, through the use of a new technique, the efficacy of the injection of BTX-A to the maxillary and mandibular nerves was evaluated.

**Methods:**

A total of 27 patients were injected with 100 Units of BTX-A to the maxillary and mandibular nerves. Visual analogue scale score and pain frequency were assessed before treatment and at the first week, second month, and sixth month after treatment. Patients with ≥50% reduction in mean pain score at the second and sixth month were defined as responders.

**Results:**

A total of 27 patients were included in the study. BTX-A significantly reduced pain intensity and pain attack frequency at the first week, second month, and sixth month after treatment. At the second month, 74.1% of patients, at the sixth month, 88.9% of patients responded to treatment. Forty-four percent of patients did not experience any pain at the sixth month. The mean recurrence period was 87.7 ± 20.4. BTX-A was well tolerated and showed few treatment-related adverse events.

**Conclusion:**

Injection to the maxillary and mandibular roots seems to be a highly effective method. In the event of recurrence, after each injection, the pain severity and attack frequency decreased.

## 1 Introduction

Trigeminal neuralgia (TN) is defined as sudden, usually unilateral severe brief stabbing recurrent episodes of pain within the distribution of one or more roots of the trigeminal nerve. The prevalence of the disease is 4 to 29 per 100,000 in the worldwide population.^[1–3]^ Oral antiepileptic drugs, including carbamazepine, remain the first line of treatment.^[4]^ Yet, 25% to 50% of cases become refractory to the drug therapy.^[5]^ Surgical intervention is occasionally used to treat severe and often untreatable TN as it can cause complications that may be worse than at the primary point.^[6]^ Although the therapeutic effect of botulinum toxin type-A (BTX-A) has been reported, it has not been widely used in the treatment of TN.

Onabotulinum toxin A is of the serotype (A, B, C_1_, C_2_, D, E, F, and G) of botulinum neurotoxin.^[7]^ It was reported to be effective for cases of TN.^[8–12]^ In these studies, BTX-A was mostly administered intradermally and subcutaneously.

We carried out this study to assess the efficacy and safety of BTX-A injected to the maxillary nerve around the pterygopalatine ganglion and mandibular nerve around the trigeminal ganglion area in patients with TN.

## 2 Material methods

The trial was approved by the local ethics committee of Istanbul Kartal Dr. Lütfi Kirdar Training and Research Hospital. The goal, procedure, and safety aspect of the study was explained to every patient before the treatment.

This study included patients who were referred to the Neurology polyclinic and diagnosed with classical TN between April 2006 and September 2016. Although most patients have been followed-up until now, this study included the patients’ first 6-month follow-up after BTX-A treatment. Baseline examinations were performed before the start of the study. The patients were examined at the first week, at the second month, and at the sixth month. During this period, patients were asked to contact the hospital if the pain recurred. In a recurrence situation, patients were examined again and injections were repeated when they were required.

Each patient underwent magnetic resonance imaging to rule out the presence of structural pathology. According to the International Classification of Headache Disorders (ICHD-2), they were diagnosed with classical TN.^[13]^ Before treatment, patients’ demographics, age, gender, presence of trigger factors, side of involvement, and duration of the disease and drugs were also recorded. At the baseline, most patients had been using medications, including carbamazepine, gabapentine, pregabalin, and amitriptyline. These medications were stopped and no new medications were started during the study periods.

Entry criteria for the study were failure of the current treatment; baseline pain intensity should be ≥4; and attack frequency should be ≥4 per day. Exclusion criteria for the study included any systemic disease or usage of any agents that might interfere with BTX-A. Women who were pregnant or planning pregnancy during treatment were excluded.

Each vial of Allergan BOTOX (onabotulinum toxin A) contains 100 Units (U) of *Clostridium botulinum* type A neurotoxin complex, 0.5 mg of human albumin, and 0.9 mg of sodium chloride. The content was diluted in 2 mL saline solution (0.9%). For each root 50 U (1 mL) was injected.

### 2.1 Injection technique

We used a dental needle of 0.40 × 50 mm for injection. For the injection to the maxillary root, through the upper edge of the zygomatic arch, patients were in a sitting position and their heads were supported by a headrest. At the upper edge of the zygomatic arch, midway between the external ear and the orbital rim, the needle was pointed toward the zygomatic bone on the other side of the skull (forming obtuse angles to the front and below) at a depth of 50 mm around the pterygopalatine ganglion. For the injection to the mandibular root, through the lower edge of the zygomatic arch, the position was the same. Their mouths were slightly open. The needle was pointed transversely along the base of the skull toward the middle and inserted below the middle of the zygomatic arch. After striking the pterygoid process, the needle was withdrawn slightly and rotated craniodorsally about 5 to 10 mm and the solution was administered around the trigeminal ganglion.

**Figure 1:**
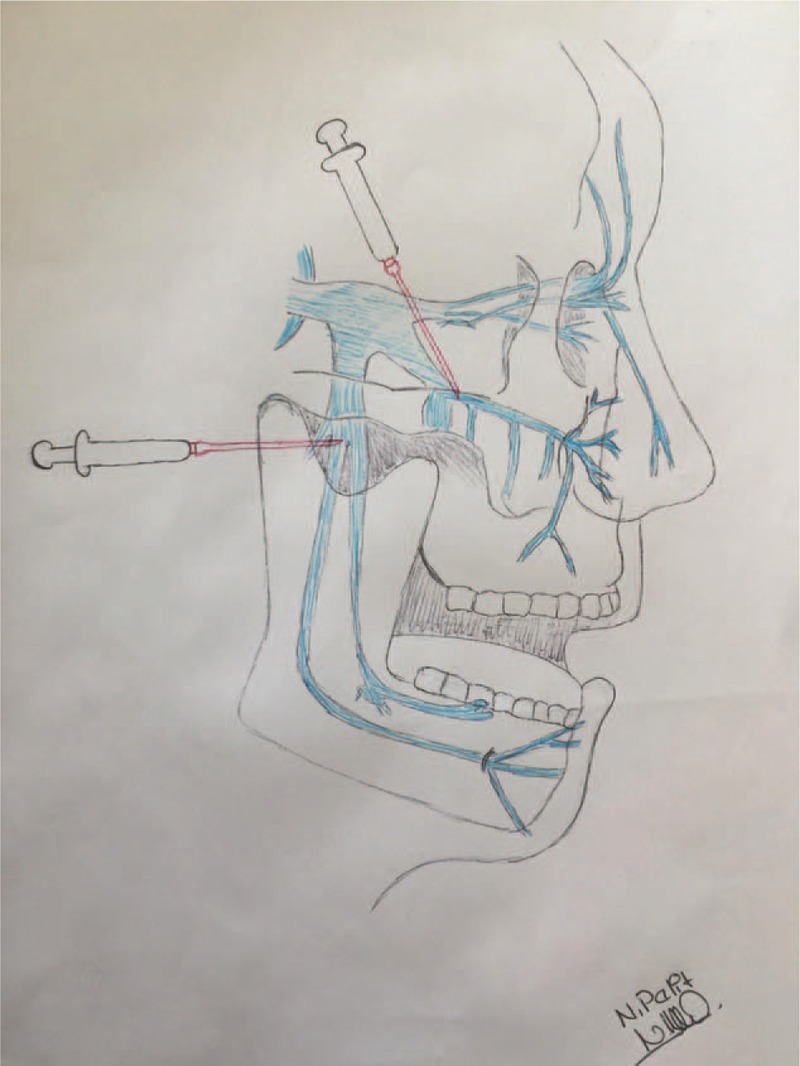
A radioscopic or echographic guide was not used during the procedure. Anatomical picture is shown in Figure 1.

A radioscopic or echographic guide was not used during the procedure. Anatomical picture is shown in Figure 1.

### 2.2 Efficacy and safety measures

The severity of pain was evaluated through the visual analogue scale (VAS) (according to an 11-point visual analogue score). VAS and pain frequency were recorded just 24 hours before the treatment, at the first week, the second month, and the sixth month. If pain recurred, the same treatment was repeated and then VAS and pain frequencies were recorded.

The overall response to treatment was assessed through the Patients Global Impression of Change (PGIC) scale. The PGIC is a self-evaluation of the patients overall change since the beginning of the study according to a 7-point scale (1, very much improved; 2, much improved; 3, minimally improved; 4, no change; 5, minimally worse; 6, much worse; and 7, very much worse). Response to treatment was defined as patients with ≥50% reduction in mean pain score from baseline to end point. The adverse events were recorded at each visit.

Statistical analyses were performed using PASW Statistics 18.0 software. Frequency distributions and percentages were calculated. Repeated measures were compared by means of the Wilcoxon signed-rank test. Response rates at different time periods were compared with the McNemar test. Results were considered to be statistically significant at the level of *P* < .05.

## 3 Results

A total of 27 patients were included in the study. Their ages ranged from 27 to 77 years (the mean age being 54.8 ± 4.5). There were 6 males (22%) and 21 females (78%). The mean duration of the disease was 4.2 ± 2.6 years. One root was affected in 10 patients whereas 2 roots were affected in 17 patients.

The mean baseline pain score (VAS) was 9.7 ± 0.6. It was found to be 3.5 ± 3.2 at the first week, 2.4 ± 3.1 at the second month, and 1.6 ± 2.4 at the sixth month. There were significant differences between the baseline VAS score and the first week score, the second month score, and the sixth month score (*P* = .000, *P* = .000, and *P* = .000, respectively). Attack frequency was calculated as the number of attacks per day. The mean baseline attack frequency was 217.7 ± 331.5, it was found to be 71.5 ± 196.3 at the first week, 54.8 ± 196.3 at the second month, and 55.15 ± 196.20 at the sixth month. There was a statistically significant difference between baseline frequency and the frequency at the first week, the second month, and the sixth month (*P* = .000, *P* = .000, and *P* = .000, respectively) (Table 1).

**Table 1:**
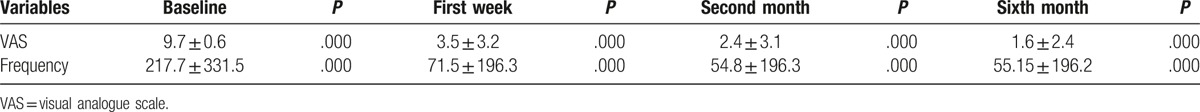
Visual analogue score (VAS) and frequency changes.

**Figure 2:**
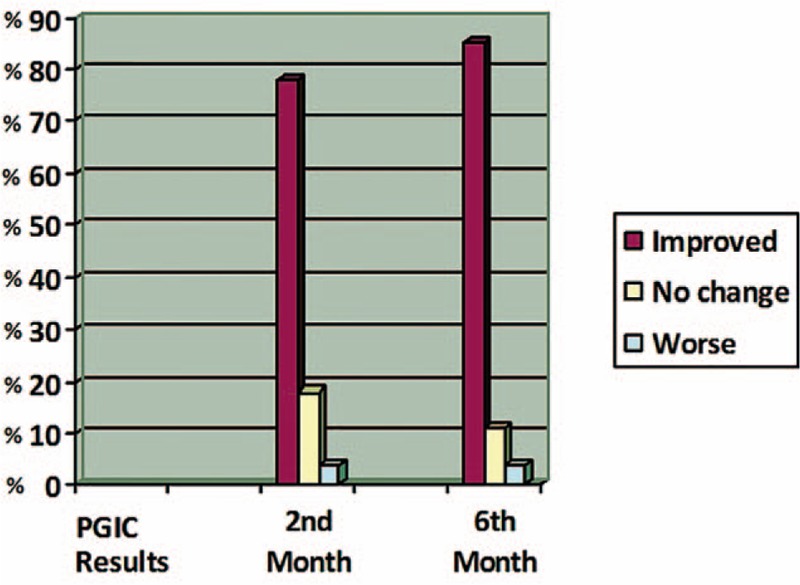
The results of PGIC. PGIC = Patients Global Impression of Change.

Evaluation of PGIC: 15/27 (55.5%) of patients reported very much improved or much improved pain score at the first week, 21/27 (77.7%) of patients reported a very much improved or much improved pain score at the second month, and 23/27 (85.1%) of patients reported a very much improved or much improved pain score at the sixth month (Figure 2).

Response to the treatment was defined as a ≥50% decrease in pain score from baseline to end point. Based on this criteria, at the first week 17/28 (63%), at the second month 20/27 (74.1%), and at the sixth month 24/27 (88.9%) of patients responded to the treatment. Out of 27, 12 (44.4%) patients were pain-free at the end of 6 months. Out of 27, 15 (55.5%) patients experienced recurrence after a mean period of 54.7 ± 30.5 (9–97) days, and they were treated for a second time. Out of 15, 7 (47%) patients experienced a second recurrence after a mean of 87.7 ± 20.4 (59–120) days. Despite being given a third injection over the 6-month period, 1 patient did not experience any change whatsoever in pain frequency and severity.

Adverse events: 1 patient experienced short-term facial weakness on the injection side, this side effect disappeared within 2 months. Masseter weakness on the injection side was observed in 2 patients, and after the third injection, remained mild and permanent.

## 4 Discussion

These results indicated that patients receiving BTX-A experienced statistical and clinical improvement in pain intensity and attack frequency at each follow-up.

In this study, it was found that the VAS score significantly decreased at the first week and continued to decrease more and more at the second month, and at the sixth month. A recurrence was not observed in around half of the patients at the sixth month. In the remaining half, recurrence developed. The recurrence period varied between 9 and 120 days. The follow-up of patients is still ongoing. We also aim to publish the long-term results of the study. As it stands, 2 patients have been recurrence-free for 2 years. The pain severity and attack frequency decreased more and more after each injection. These patients are continually being followed-up. In this study, we did not use the same technique as other authors. This technique showed that pain severity decreased by almost 90% at the sixth month.

BTX-A in TN has been used over the last decade, almost in all studies BTX-A was injected subcutaneously, intracutaneously, and into the trigger zone in the painful facial area.^[9,12,14–16]^ In our previous study, we injected 50 U of BTX-A above the zygomatic arch and 50 U of BTX-A below the zygomatic arch. It showed a 70% decrease of mean VAS score at the sixth month.^[10]^

Wu et al reported that BTX-A for the treatment of TN was effective and safe, they injected intradermally and submucosally where pain was experienced. Their response rate was also high at 70% at week 8.^[14]^

Jian-Huo Xia et al treated 86 patients with TN; they also injected intracutaneously in the painful facial area. Their efficacy rate was 80% at the eighth week. Other studies reported that BTX-A injection in the facial pain area significantly relieves pain in TN.^[15,16]^

Batifol et al have recently found that the injection into the trigger zone required considerably lower doses. The effectiveness of the BTX-A showed high response rate in their study.^[17]^

Sheta et al reported in their single-blinded randomized placebo-controlled study that there was significant pain reduction in the BTX-A group when compared to placebo group. There was also a significant decrease in the number of acute medication and an increase in quality of life.^[18]^

Zhang et al reported in their double-blinded randomized placebo-controlled trial, administered 25 U to one group and 75 U to another and compared with a placebo group. Results showed that the response rates were significantly higher in BTX-A groups than placebo group, but there was no statistically significant difference between 25 U and 75 U groups.^[19]^

Evidence from systematical review showed that only 4 randomized controlled studies were undertaken up to the 2016. In total, 176 TN patients were treated. According to their results, BTX-A is a significantly effective and beneficial substance in the treatment of TN when compared with use of a placebo. Their injection technique was subcutaneous and/or intradermal.^[20]^

With our injection technique, the injection was administered in close proximity to the maxillary and mandibular nerve roots around the ganglions. We observed 88.9% success rate with this method. These results were slightly more effective than the other methods.

No significant safety concerns were recorded in this study. Masseter weakness was noticed by the patient after detailed questioning. Despite this adverse event, the patients wanted to continue with injections due to satisfaction.

### 4.1 Limitations of the study

Most important limitation of this study is that it is not a placebo-controlled study. Second limitation, the fact that our study is an open label study may create bias.

The mechanism of onabotulinum toxin A in controlling pain in TN remains uncertain. Several studies have been conducted to explore the mechanism underlying the potential analgesic action of BTX-A. After being injected in the subcutaneous tissue, BTX-A is taken up by endocytosis at nerve terminals of C fibers and rises by retrograde axonal transport through the trigeminal ganglion to the spinal trigeminal nucleus.^[21]^ One of the main antinociceptive effects of BTX-A is probably related to its ability to block the transport of nociceptive input to centers modulating nociception.^[22]^ BTX-A negatively modulates nociceptive neurotransmitters. Its action can be preganglionic, on CGRP,^[23–25]^ substance P^[26]^ and glutamate,^[27]^ or postganglionic, on synaptic terminations, blocking the release of norepinephrine (NE) and adenosine triphosphate (ATP).^[28,29]^

In conclusion, this study showed that this injection technique seems to be a highly effective method. In the event of recurrence, a repeat of injections should not be avoided. Evidence of longer and well-designed and randomized controlled trials is required.
